# A case of disseminated cryptococcosis with necrotizing fasciitis in a non‐HIV patient

**DOI:** 10.1002/ams2.298

**Published:** 2017-07-13

**Authors:** Tetsuya Hoshino, Kazuya Omura, Shinichi Kimura, Hiroyuki Takahashi, Katsuhiko Kamei, Misako Ohkusu

**Affiliations:** ^1^ Department of Intensive Care Medicine Saiseikai Yokohamashi Tobu Hospital Yokohama Kanagawa Japan; ^2^ Division of Clinical Research Medical Mycology Research Center Chiba University Chiba Japan

**Keywords:** Cryptococcal meningitis, disseminated cryptococcosis, HIV‐negative, necrotizing fasciitis, steroid

## Abstract

**Case:**

Disseminated cryptococcosis is a well‐recognized condition among HIV patients, but it also occurs in non‐HIV patients. Necrotizing fasciitis caused by cryptococcus is rare. An 81‐year‐old man who had received steroid therapy presented with erythema and pain in his right thigh. After the rapid progression of symptoms and a failure to respond to antibiotic therapy, a clinical diagnosis of necrotizing fasciitis was made. We performed debridement, and yeasts were detected using a Gram stain of the fascia. We treated the patient with liposomal amphotericin B. On day 3, he developed meningitis. *Cryptococcus neoformans* was detected in the blood, fascia, and cerebrospinal fluid. Flucytosine was added to liposomal amphotericin B.

**Outcome:**

Despite the antifungal treatment, new regions of dissemination to the skin developed, and the patient died of multiple organ failure.

**Conclusion:**

A diagnosis of disseminated cryptococcosis should be considered in a differential diagnosis of necrotizing fasciitis among immunocompromised patients, regardless of their HIV status.

## Introduction

Disseminated cryptococcosis (DC) is a well‐recognized condition among patients infected with HIV, but it can also occur in non‐HIV patients. Cryptococcal infection commonly presents as meningitis. However, infection of the skin and other organs may also be involved in disseminated cryptococcosis. Cutaneous infections are reported in 10–20% of patients.^1^ Primary cutaneous infections are rare, and cutaneous manifestations are often signs of disseminated disease. Dermatological manifestations of cryptococcal infections include ulcers, abscess, granulomas, and pustules. Necrotizing fasciitis (NF) is rare.^2^

## Case

An 81‐year‐old man with a medical history of chronic kidney disease and diabetes mellitus (DM) who had been receiving steroid therapy (dexamethasone for 3 years, 1 mg/day, for prostate cancer; prednisolone for 10 years, 2.5 mg/day, for purpura nephritis) presented with a right thigh pain beginning 3 months prior to admission and progressing to erythema, swelling, and a worsening pain 2 weeks before admission. He had been admitted to another hospital, and antibiotics had been given. However, his symptoms progressed and he was diagnosed as NF. Therefore, he was transported to our hospital. He had not been exposed to pigeon excrement. At the time of admission, his consciousness was clear. His pulse rate was 100 b.p.m., his respiratory rate was 24 breaths/min, his blood pressure was 130/93 mmHg, his body temperature was 37.1°C, and his O_2_ saturation was 98% (ambient room air). On physical examination, erythema and swelling were seen in his right thigh. His laboratory results were as follows: white blood cells, 13.2 × 10^9^/L with 12.8 × 10^9^/L neutrophils and 0.20 × 10^9^/L lymphocytes; hemoglobin, 11.0 g/dL; platelets, 46.7 × 10^9^/L; creatinine‐phosphokinase, 37 IU/L; serum creatinine, 4.09 mg/dL; and C‐reactive protein, 22.1 mg/dL. Serology testing for HIV was negative.

**Figure 1:**
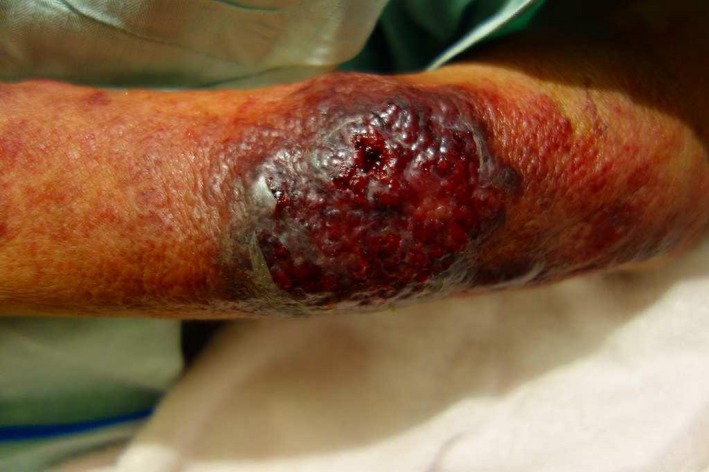
Disseminated skin region in an 81‐year‐old man with disseminated cryptococcosis with necrotizing fasciitis. Epidermal necrosis and a hematoma appeared on the patient's right arm.

**Figure 2:**
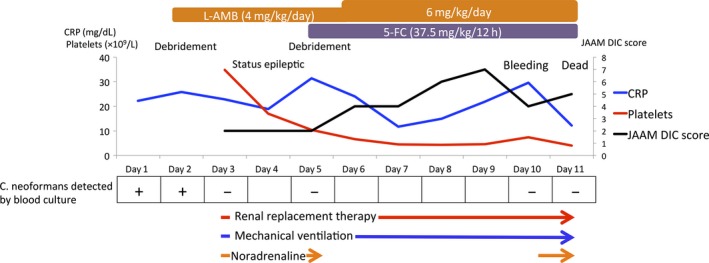
Clinical course of an 81‐year‐old man with disseminated cryptococcosis with necrotizing fasciitis following admission to the intensive care unit. 5‐FC, flucytosine; *C. neoformans*,* Cryptococcus neoformans*. CRP, C‐reactive protein; JAAM DIC, Japanese Association for Acute Medicine Disseminated Intravascular Coagulation; L‐AMB, liposomal amphotericin B.

Surgical debridement was carried out on day 2. Yeasts were detected using a Gram stain of the fascia. Treatment with liposomal amphotericin B (L‐AMB) 4 mg/kg/day was initiated. On day 3, he presented with septic shock and a disturbance of consciousness and was moved to the intensive care unit (ICU). At the time of ICU admission, his Glasgow Coma Scale score was 3 (E1V1M1). The findings at the surgical site did not change, but a new skin lesion appeared on the patient's right arm (Fig. 1). He was intubated and mechanically ventilated. A lumbar puncture was carried out on day 3. The patient's cerebrospinal fluid (CSF) pressure was low (<5 cmH_2_0). The CSF was found to be clear with a cell count of 5/μL and protein and glucose levels of 60 and 50 mg/dL (serum glucose 130 mg/dL), respectively. Yeast cells with thick capsule were cultured from a blood sample, the fascia of the right thigh, and the CSF, which were later identified as *Cryptococcus neoformans* with ID32C API system. The identification of the fungus was confirmed as *C. neoformans* serotype A by the analysis of the *IGS* gene along with multiplex polymerase chain reaction amplification of *LAC1* and *CAP64* genes.^3^ The patient was diagnosed as having disseminated cryptococcosis. Flucytosine 37.5 mg/kg/12 h was added to L‐AMB. On day 5, debridement of the right arm was carried out and *C. neoformans* was cultured from a tissue sample. On day 10, however, purpura occurred on the patient's left leg. Venous bleeding from the wound surface on the right thigh occurred, resulting in hemorrhagic shock. Blood transfusion was required. Hemostasis was obtained, but the hemodynamic status remained unstable. A laboratory test on day 11 showed hepatic failure (aspartate aminotransferase, 1700 U/L; alanine aminotransferase, 253 U/L; prothrombin time – international normalized ratio, 1.77; platelets, 4.0 × 10^9^/L). The patient died on day 11 from multiple organ failure (Fig. 2).

## Discussion

In Japan, all cases of DC must be reported. In total, 126 cases were reported between September 2014 and August 2015, and only eight cases were HIV patients.^4^ In 2015, 1,434 cases were reported as newly infected with HIV. The number of new cases of HIV infection has been gradually increasing in Japan.^5^ Human immunodeficiency virus is well recognized as a risk factor for DC, but the number of HIV patients with DC is relatively small in Japan. However, the incidence of DC in HIV patients could potentially increase.

Other risk factors for DC include immunosuppressive medicines, steroid therapy, malignancy, solid organ transplantation, renal insufficiency, cirrhosis, DM, and hematologic disorders.^6^

The current recommendation for antifungal treatment for Cryptococcus is amphotericin B plus flucytosine in non‐HIV, non‐transplant hosts. In cases with amphotericin B intolerance, L‐AMB is recommended as a substitute for amphotericin B.^7^

The prognosis of non‐HIV patients with DC is relatively poor, compared with HIV patients. The 1‐month mortality rate has been reported to be 63%, and 81% of the deaths occurred within 2 weeks of diagnosis.^8^

In a review of published reports, we identified seven cases of DC with NF (Table 1). Most of these patients were on long‐term steroid treatment after organ transplantation. The most common infection site was the lower extremity. The mortality rate of these cases was 57% (4/7). All the survival cases had undergone surgical debridement and DC was the systemic infection. However, the focus on control by surgical debridement is necessary for treatment of DC with NF.

**Table 1:** Characteristics of published cases of disseminated cryptococcosis with necrotizing fasciitis

Author/country/year	Age, years/sex	Cryptococcus variety	Comorbidities	Immunosuppressant	Infection site	Antifungal	Surgical debridement	Outcome
Marcus^9^/ USA/1998	43/M	Neoformans	Renal transplant	Cyclosporine 150 mg twice daily, prednisolone 10 mg daily	Bilateral thighs	AMB	+	Alive
Huang^10^/ Taiwan/2007	58/M	Neoformans	DM	NI	Right thigh	AMB	+	Death
Capoor^2^/ India/2008	40/M	Neoformans	Chronic alcoholism	NI	Right gluteus	AMB	+	Alive
Baer^11^/ USA/2009	61/M	Neoformans	Renal transplant	Cyclosporine 50 mg twice daily, MMF 750 mg twice daily, prednisolone 10 mg daily	Bilateral calves	L‐AMB	+	Alive
	64/M	Neoformans	Renal transplant	MMF 750 mg twice daily, Tacrolimus 1 mg twice daily, Prednisolone 10 mg daily	Left leg	Fluconazole	−	Death
	57/M	Neoformans	Heart transplant	MMF 750 mg twice daily, prednisolone 10 mg daily	Bilateral calves	L‐AMB + 5‐FC	−	Death
Hoshino/Japan/2017	81/M	Neoformans	DM, prostate cancer, CKD	Dexamethasone 1 mg daily, prednisolone 2.5 mg daily	Right thigh	L‐AMB + 5‐FC	+	Death

5‐FC, flucytosine; AMB, amphotericin‐B; CKD, chronic kidney disease; DM, diabetes mellitus; L‐AMB, liposomal amphotericin‐B; M, male; MMF, mycophenolate mofetil; NI, no information.

Cryptococcal NF in non‐HIV patients is very rare; therefore, the reporting of this case is valuable. The presently reported patient had renal insufficiency, steroid use, DM, and prostate cancer. Therefore, he had a high risk of DC with NF. Because no deficits in the skin barrier were apparent, we assumed that the pathway of infection was the respiratory tract. Because of the disruption of immune control, *C. neoformans* was able to disseminate to the skin, soft tissue, and CNS.

After ICU admission, the patient's hemodynamic status improved following the initial resuscitation. The physical findings for his right thigh did not show signs of exacerbation. Blood cultures on days 5, 10, and 11 were all negative. However, he presented with a new region of dissemination to the skin, and a bleeding event occurred in association with DIC. Insufficient control of the infection and hemorrhagic shock resulted in multiple organ failure. We could not rescue him, despite antifungal treatment and surgical debridement. To improve the prognosis of cryptococcal NF, early diagnosis and early treatment are necessary. Disseminated cryptococcosis should be considered in a differential diagnosis of NF in immunocompromised patients regardless of their HIV status.

## Conclusion

We experienced a case of DC with NF in a non‐HIV patient. A diagnosis of DC should be considered in a differential diagnosis of NF among immunocompromised patients, regardless of their HIV status. Early diagnosis and early treatment are necessary to improve the outcome.

## Disclosure

Conflict of interest: None.

Approval was obtained from the appropriate Institutional Review Board (2016089).
